# 363. Characteristics of Envelope and Nuclear Gene Expression Patterns in Asymptomatic SARS-CoV-2 Patients: A Single Center Retrospective Observational Study

**DOI:** 10.1093/ofid/ofab466.564

**Published:** 2021-12-04

**Authors:** Keiko Mizuno, Takayuki Komatu, Daisuke Usuda, Shiori Hocchi, Karin Ashizawa, Takashi Aoki, Kanako Ogura, Hiroki Takami, Tomohisa Nomura, Manabu Sugita

**Affiliations:** Juntenodo University Nerima Hospital, Nerima, Tokyo, Japan

## Abstract

**Background:**

Reverse transcription-polymerase chain reaction (RT-PCR) is used for the diagnosis of COVID-19, caused by SARS-CoV-2. RT-PCR is a method that detects the virus by amplifying two regions of the target viral genome, namely the nuclear (N) and envelope (E) encoding sequences. However, no reports have shown a relationship between the symptoms and the gene expression patterns, especially in asymptomatic patients. Herein, we validated the characteristics of E and N gene expression patterns using RT-PCR on samples obtained from asymptomatic COVID-19-positive patients.

**Methods:**

In this retrospective cohort study, conducted at Juntendo University Nerima Hospital, Tokyo, Japan, SARS-Cov-2 RT-PCR positive patients whose specimens had been obtained and analyzed by our laboratory technicians from September 1, 2020 to December 31, 2020 were enrolled. For RT-PCR, the LightMix Modular SARS-CoV-2 reagent (TIB MOLBIOL company) was used. After excluding patients who had symptoms, background, demographic, laboratory, and gene expression pattern data were collected from RT-PCR-positive asymptomatic patients. We also investigated patients who met the release criteria of the Center for Disease Control and prevention. Continuous and categorical variables were analyzed, with p< 0.05 set as statistical significance using the student-t test, chi-square test, or Fisher’s exact test, respectively.

**Results:**

Of 92 RT-PCR-positive asymptomatic patients, 57 comprised the expression E only group (Group E) and 35 comprised the E+N group (Group E+N). Significantly more patients in Group E met the release criteria compared to those in Group E+N [41 (71%) vs 10 (28%), *p*< 0.001]. Among patients who met the release criteria, those in Group E+N had significantly more immunosuppression [7 (70%) vs 8 (30%), *p*=0.004].

Moreover, among the patients who underwent RT-PCR screening, no patients in Group E developed symptoms [0 vs 6 (42%), *p*=0.02].

**Conclusion:**

The results of this study suggest that RT-PCR-positive asymptomatic patients can be divided into three patterns: pre-symptomatic, gene E+N-positive patients; post-symptomatic covid-19-recovered patients, regardless of gene E and N expression patterns; and false positive, gene E-positive patients.

**Disclosures:**

**All Authors**: No reported disclosures

